# Transperineal prostate biopsy with freehand technique under local anaesthetic: A systematic review and meta‐analysis

**DOI:** 10.1002/bco2.70016

**Published:** 2025-04-08

**Authors:** Benjamin M. Mac Curtain, Gavin Calpin, Josh Bruinsma, Wanyang Qian, Avinash Deshwal, Eoin Collins, Hugo C. Temperley, Reuben D. Mac Curtain, William P. Shields, Lee Chien Yap, Claudiu Cozman, John Keane, Padraig Daly

**Affiliations:** ^1^ Royal College of Surgeons in Ireland Dublin Ireland; ^2^ Department of Urology University Hospital Waterford Waterford Ireland; ^3^ Department of Urology St Vincent's University Hospital Dublin Ireland; ^4^ Department of Urology Sir Charles Gairdner Hospital Perth Western Australia; ^5^ Department of Surgery St John of God Midland Hospital Perth Western Australia; ^6^ Department of Surgery Fiona Stanley Hospital Perth Western Australia; ^7^ Department of Radiation Oncology Cork University Hospital Cork Ireland; ^8^ Department of Radiology St James's Hospital Dublin Ireland; ^9^ School of Medicine University College Dublin Dublin Ireland

**Keywords:** CAMProbe, freehand, local anaesthetic, precision point, prostate cancer, Transperineal prostate biopsy

## Abstract

**Background:**

Transperineal prostate biopsy (TPPB) under local anaesthesia is a widely employed biopsy method, and is currently endorsed by the European Association of Urology (EAU). This review aimed to assess the pooled detection rates of clinically significant prostate cancer using TPPB under local anaesthetic. Additionally, pain scores and complications were also reported.

**Methods:**

Our search was conducted in line with the most recent Preferred Reporting Items for Systematic reviews and Meta‐Analyses (PRISMA) recommendations up to August 2024. The study was registered on PROSPERO under the ID: CRD42024588824. An electronic search was conducted of the PubMed, Embase and Cochrane Central Register of Controlled Trials databases along with grey literature using the Google search engine.

**Results:**

In total, there were 2881 patients included in this review. Biopsy histology results were reported in 11 studies comprising 2781 cases. We observed a clinically significant prostate cancer rate of 52% (95% CI 44%–60%) for studies that employed both a mix of systematic and targeted biopsies and 26% (95% CI 23%–30%) when systematic biopsies alone were taken. The pooled rate was 48% (95% CI 37%–59%), overall. Complications after prostate biopsies were reported by 9 studies with a combined 2688 patients. There were 61 patients (2.3%) who had Clavien–Dindo (CD) 1–2 complications and three patients (0.1%) who had CD 3–5 complications. The pooled rate of CD 1 and 2 complications was 2% (95% CI 1%–4%).

**Conclusions:**

TPPB under local anaesthetic is a safe, efficacious and well‐tolerated method of prostate biopsy when compared with other methods. Undertaking the procedure under local anaesthesia does not seem to lower cancer detection rates.

## 1 INTRODUCTION

Prostate cancer (PCa) is the most commonly diagnosed cancer in men and is the sixth most common cause of cancer mortality. It has been reported that 359 000 men died as a result of prostate cancer in 2018. This is only expected to grow secondary to the ageing population across the world.^1^

No difference has previously been shown in cancer detection rates considering general and local anaesthesia use in transperineal prostate biopsy (TPPB).^2^ There are a number of anaesthesia block methods available for use at the discretion of the urologist or radiologist performing the biopsy, each with various reported visual analogue scale results.^3^ Overall, it has been reported in the literature as a well‐tolerated procedure under freehand technique and local anaesthetic.^4^ The procedure has been previously described using the Ginsburg protocol sampling technique, which is one of many sampling methods.^5^

TPPB without the use of antibiotics has been described as having equivocal infectious complications compared with transrectal (TRUS) biopsy with antibiotic prophylaxis without rectal swabs.^6^ Evidence suggests TPPB with or without antibiotics carries a low risk of sepsis and infection post biopsy.^7^ These results are echoed in the PREVENT trial conducted by Hu et al. Again, they showed TPPB without antibiotic prophylaxis did not have any statistically significant difference in infection complications. However, there were no infections in the TPPB group compared with four in the TRUS group. The *p* value of 0.059 associated with this comparison shows this difference did not reach statistical significance in initial results.^8^ However, at full follow‐up, the infection rates were observed to be statistically significantly lower in the TPPB group.^9^

TPPB pain scores within 7 days of the biopsy were slightly less favourable compared with TRUS biopsy. Clinically significant prostate cancer (CSPC) rates in the TPPB group were slightly higher, with no statistically significant difference observed.^8^ Similar infectious complications were observed relating specifically to freehand TPPB.^10^ There is evidence that TPPB combined with prebiopsy MRI is safe and detects satisfactory levels of CSPC.^11^

TPPB is not only advantageous in terms of a low risk of infection post procedure; it has potential anatomical access benefits in comparison to TRUS biopsy and may provide superior sampling capacity for anterior and apical tumours.^12^ In addition to the clinical benefits, double free hand TPPB has been reported as a cost‐effective technique.^13^ Taking all the evidence into account, the widespread adoption of TPPB in the United States has been advocated, especially with the inclusion of preoperative MRI.^14^, ^15^

A previous comprehensive systematic review and meta‐analysis was undertaken by Kanagarajah et al including papers up to early 2021.^16^ Tzeng et al also published a comprehensive meta‐analysis in 2022 reporting a CSPC rate of 43%; however, pain scores were not reported.^17^ A number of important landmark trials have recently been published since then as mentioned above prompting a re‐review of the topic. This review provides a contemporary synthesis and statistical summary of the current evidence in relation to TPPB under local anaesthetic and freehand technique to ensure similar PCa detection rates to reported values and tolerability.

## 2 METHODS

### 2.1 Registration and search strategy

Our search was conducted in line with the most recent Preferred Reporting Items for Systematic reviews and Meta‐Analyses (PRISMA) recommendations.^18^ The study was registered on PROSPERO under the ID: CRD42024588824. An electronic search was conducted of the PubMed, Embase and Cochrane Central Register of Controlled Trials databases utilising the search algorithms provided below up to 24/8/2024. One study was added to our search database manually from the grey literature.^2^

(transperineal prostate biopsy or transperineal biopsy or tp biopsy) and (freehand or free‐hand or free hand) and (local anesthetic or local anesthesia or regional anesthetic or regional anesthesia or local anaesthetic or local anaesthesia or regional anaesthetic or regional anaesthesia) and (outcomes or pain or pain score or rates or clinically significant prostate cancer)

**FIGURE 1:**
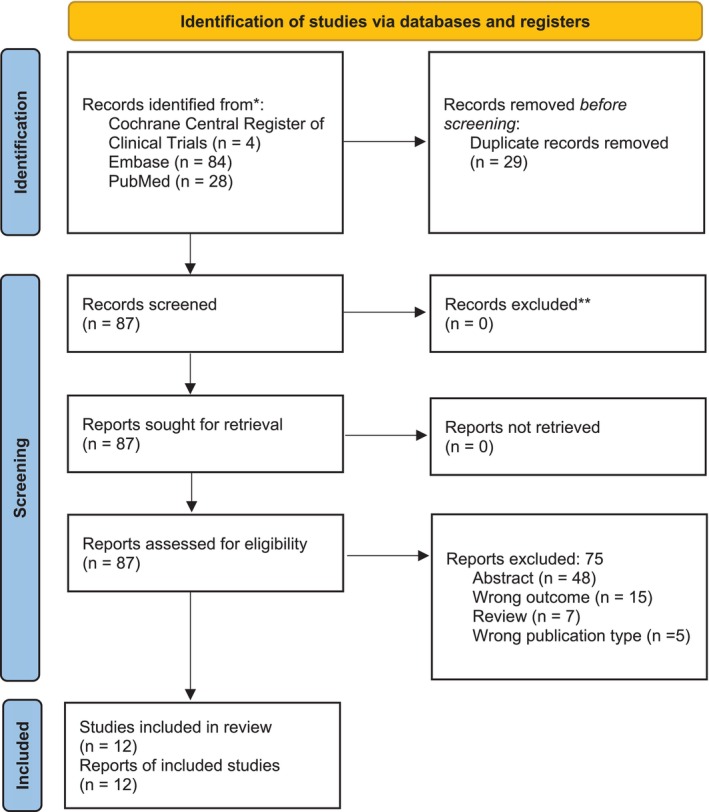
PRISMA flowchart.

The complete breakdown of analysed studies can be viewed in the PRISMA diagram in Figure 1. The bibliographies of included publications were also searched for any relevant studies.

Inclusion criteria were as follows:

- Studies detailing the use of TPPB under local anaesthesia with or without sedation.
- Reports rates of CSPC or complications or pain scores.
- Any form of local anaesthetic technique.
- Prostate biopsy needle and sheath may be completely free in space or mounted to the rectal ultrasound probe, but it should be a ‘freehand technique’.
- Targeted (cognitive fusion only) and systematic, or both, biopsy types were considered.
- Retrospective or prospective studies.
- Full text in English language available.

Exclusion criteria were as follows:

- Nonadherence to inclusion criteria.
- Case series defined as 10 or less participants, case reports, conference abstracts.
- TRUS biopsy or general anaesthesia studies.
- Studies employing the use of US/MRI fusion software for targeting.
- Robotic prostate biopsy systems.

### 2.2 Identification of studies and outcomes of interest

Studies that satisfied the inclusion and exclusion criteria were included. The following PICO elements were used as the basis for selecting studies^19^:

*Population*: Men undergoing prostate biopsy.

*Intervention*: TPPB under local anaesthesia with freehand technique.

*Comparison*: TPPB performed under general anaesthetic or TRUS biopsy.

*Outcome*: Rate of detection of CSPC or pain score/complications.

Studies were independently reviewed by two separate authors (BMC, GC) using Rayyan.^20^ If there was any disagreement between authors, an alternative author (WQ) was used to mediate the discussion and consensus was reached.

The primary outcomes of interest were rates of CSPC and pain scores. These were chosen due to the concern that pain may limit sampling and lead to a noncomprehensive sampling profile. As alluded to in the Introduction, pain scores were seen to be higher in the TPPB group when compared with a TRUS cohort up to days post procedure.

Secondary outcomes comprised the rates of complications. The study by Ashouri et al was not included in this review's rates of prostate cancer as a micro ultrasound technique was used.^23^

### 2.3 Data extraction

Study demographics and biopsy variables of interest were transcribed using Google Sheets (Mountain View, California, USA). Four independent authors (JB, WQ, AD and RMC) were involved in the data extraction.

### 2.4 Study selection

Prospective and retrospective studies including randomised trials, if applicable, were included in this systematic review and meta‐analysis. Studies were selected on the basis of their fulfilment of the inclusion criteria and availably in the English Language.

### 2.5 Risk of bias assessment

Assessment of potential biases for nonrandomised studies was assessed using a modified Newcastle‐Ottawa scale risk of bias tool,^21^ with the results tabulated in Table 1. This assessment tool grades each study as being ‘satisfactory’ or ‘unsatisfactory’ across various categories. We assigned stars to evaluate study quality: 7 stars = *very good*, 5–6 stars = *good*, 3–4 stars = *satisfactory* and 0–2 stars = *unsatisfactory*. The critical appraisal was completed by two reviewers independently (EC and JB), where once again a third reviewer (BMC) was asked to arbitrate in cases of discrepancies in opinion.

**TABLE 1:** Study demographics.

Author	Country	Journal	Study type	*N*	Age ± SD
Honoré 2024^22^	Norway	*BJU International*	Prospective	1028	Median 68 (IQR 63–72)
Ashouri 2023^23^	USA	*J Vis Exp*	Retrospective	100	‐
Silva 2023^24^	Portugal	*Acta Urológica Portuguesa*	Prospective	108	Mean 66 ± 9
Sivaraman 2022^25^	India	*Indian Journal of Urology*	Prospective	50	Mean 69.6 ± 7.61
Chiu 2020^26^	Hong Kong	*Prostate Cancer and Prostatic Diseases*	Prospective	611	Median 69 (IQR 65–72)
Wetterauer 2020^27^	Switzerland	*Prostate Cancer and Prostatic Diseases*	Retrospective	400	Median 66 (IQR 49–86)
Kum 2020^28^	UK	*BJU International*	Prospective	176	Mean 65 (range 36–83)
Thurtle 2018^4^	UK	*Journal of Clinical Urology*	Prospective	30	Median 71.5
DiBianco 2016^29^	USA	*Urology Practice*	Retrospective	244	Mean 66.7 ± 8.5
Ngu 2023^5^	Malaysia	*Cancer Pathogenesis and Therapy*	Retrospective	55	Mean 67.3 ± 5.7
Alnosayan 2023^30^	Saudi Arabia	*Cureus*	Retrospective	39	Mean 70.3 ± 10.1
Hogan 2021^2^	Australia	*BJUI Compass*	Prospective	40	Median 63.5 (IQR 58.5–68.5)

### 2.6 Statistical analysis

We performed a proportional meta‐analysis as part of this review.^31^ Statistical analysis was run using Stata 17 (StataCorp. 2021. *Stata Statistical Software: Release 17*. College Station, TX: StataCorp LLC). Proportions were pooled using the ‘metaprop’ function within Stata^32^; 95% confidence intervals were employed, and *p* ≤ 0.05 was considered statistically significant. Heterogeneity was reported using *I* ^2.^ ^32^ It has been put forward that *I* ^2^ values of 25%, 50%, 75% can be used to assess the degree of heterogeneity.^33^ We considered there to be a notable degree of heterogeneity if *I* ^2^ was greater than 50%. A random‐effects model was used because of evidence of significant statistical heterogeneity as well as study design heterogeneity.^34^

We meta‐analysed the rates of CSPC stratified by targeted/systematic status. We also meta‐analysed complications if they were reported in Clavien–Dindo (CD) format. This was not done for any complication over a CD 2 as only one study reported any complications of this sort.^23^ Qualitative bias assessment was conducted as proposed by Barker et al, as this is a proportional meta‐analysis.^31^

## 3 RESULTS

### 3.1 Primary outcomes

#### 3.1.1 Literature search

Overall, 116 studies were identified from the database search. After duplicates and non‐English texts were removed, there were 87 manuscripts remaining. All studies were reviewed, and 75 were excluded, leaving 12 for inclusion in the systematic review and 10 in the meta‐analysis. The search strategy and study identification are summarised in the PRISMA flow diagram as seen in Figure 1.

### 3.2 Study characteristics

There were 12 studies included in the systematic review. Seven studies were prospective, and five studies were retrospective. Publications dates ranged from 2016 to 2024. Study demographics are outlined in Table 1.

### 3.3 Biopsy and patient characteristics

In total, there were 2881 patients included in this review. Ten studies reported on both systematic and targeted biopsies. Two studies only reported on systematic biopsies.^4^, ^26^ In terms of the perineal puncture/biopsy device, CAMprobe was used in two studies,^4^, ^22^ Tru Guide in two,^23^, ^26^ Precision Point in three,^2^, ^25^, ^28^ while Magnum,^24^ a 14G needle,^29^ a 15G needle^5^ and a 16G needle^27^ were each used in one. The needle was unspecified in one study.^30^ The PSA of patients was reported with a highest median 14.2 in Ngu et al. and a highest mean value of 36.6 in Alnosayan et al.^5^, ^30^ The prostate gland volumes showed a highest median of 46.8 cm^3^ in Chiu et al. and a highest mean of 73.5 cm^3^ in Alnosayan et al.^26^, ^30^ Biopsy and patient details are outlined in Table 2 and also include the PIRADs scores and PSA density where available.

**TABLE 2:** Biopsy, imaging and patient details.

Author	Biopsy type	Systematic or Targeted biopsy	Antibiotics?	PIRADS 3	PIRADS 4–5	PSA ± SD	Gland volume ± SD	PSA density ± SD
Honoré 2024	CAMProbe	Both	2.4% TP 100% TR	213	812	Median 8 (IQR 6–12)	Median 39 (IQR 28–53)	Median 0.21 (IQR 0.14–0.34)
Ashouri 2023	TRUGUIDE	Both	No	5	52	‐	‐	‐
Silva 2023	Magnum	Both	No	‐	‐	Median 7.7	‐	‐
Sivaraman 2022	Precision Point	Both	Yes	12	36	Median 13.5 (IQR 4.2–672)	Median 45 (IQR 16–520)	‐
Chiu 2020	TRUGUIDE	Systematic	Yes	‐	‐	Median 9.9 (IQR 6.4–16.2)	Median 46.8 (IQR 33.0–67.7)	Median 0.21 (IQR 0.13–0.38)
Wetterauer 2020	16 Gauge needle	Both	44.3% No	31	235	Median 6.4 (IQR 0.3–1400)	Median 40 (IQR 6–150)	Median 0.15 (IQR 0–31.1)
Kum 2020	Precision Point	Both	Yes	‐	‐	Median 7.9 (0.7–1374)	Mean 45 (15–157)	Median 0.23 (range 0.03–27.5)
Thurtle 2018	CAMProbe	Systematic	Yes	‐	‐	Median 5.3 (Range 0.72–36.9)	‐	‐
DiBianco 2016	14 Gauge needle	Both	Yes	‐	‐	Mean 11.2	‐	‐
Ngu 2023	15 Gauge needle	Both	Not reported	‐	‐	Median 14.2 (9.5–26.0)	Median 43.7 (30–55)	Median 0.33 (IQR 0.21–0.62)
Alnosayan 2023	Unspecified Needle	Both	Not reported	5	24	Mean 36.6 ± 39.0	Mean 73.5 ± 47.5	Mean 0.49 ± 0.61
Hogan 2021	Precision Point	Both	25%—no	‐	‐	Mean 7.36 ± 4.32	Mean 51.03 ± 17.0	‐

### 3.4 Biopsy histology

**FIGURE 2:**
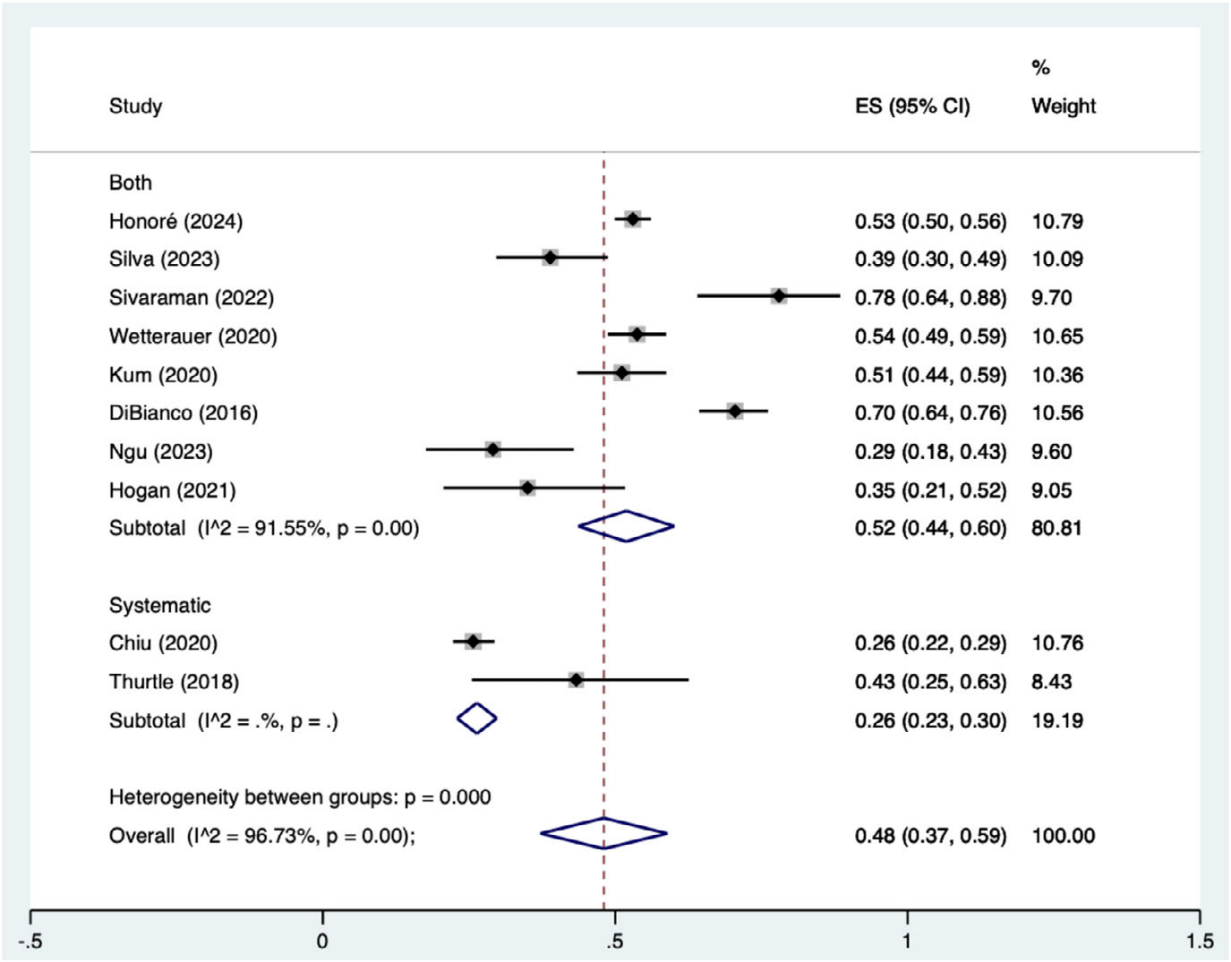
Pooled rates of clinically significant prostate cancer stratified by targeted or systematic biopsy type.

Biopsy histology results were reported in 11 studies including 2781 cases. Biopsies showed benign tissue only in 39.7% of cases (1103/2781), grade group 1 in 16.6% (450/2712) of cases and CSPC in 47.5% (1303/2742) of cases. Biopsy outcomes are outlined in Table 3. Our meta‐analysis of the pooled proportions of CSPC can be observed in Figure 2. We observe a rate of 52% (95% CI 44–60%) for studies that employed both a mix of systematic and targeted biopsies, 26% (95% CI 23–30%) when systematic biopsies are used alone and 48% (95% CI 37–59%), overall. *I* ^2^ was 96.7% and *p* < 0.05.

**TABLE 3:** Biopsy histology.

Author	Benign	GG1	GG2	GG3	GG4	GG5	Clinically significant prostate cancer (GG2 or more)
Honoré 2024	312 (30.4%)	171	297	114	51	83	545 (53.0%)
Ashouri 2023	‐	‐	‐	‐	‐	‐	‐
Silva 2023	41 (38.0%)	25	‐	‐	‐	‐	42 (38.9%)
Sivaraman 2022	9 (18.0%)	2	13	16	8	2	39 (78.0%)
Chiu 2020	381 (62.4%)	73	47	30	35	45	157 (25.7%)
Wetterauer 2020	142 (35.5%)	43	113	71	20	11	215 (53.8%)
Kum 2020	37 (21.0%)	49	‐	‐	‐	‐	90 (51.1%)
Thurtle 2018	17 (56.7%)	‐	‐	‐	‐	‐	13 (43.3%)
DiBianco 2016	102 (37.2%)	71	59	18	‐	‐	172 (62.8%)
Ngu 2023	31 (56.4%)	8	6	2	4	4	16 (29.1%)
Alnosayan 2023	13 (33.3%)	‐	‐	‐	‐	‐	
Hogan 2021	18 (45.0%)	8	‐	‐	‐	‐	14 (35.0%)

### 3.5 Post biopsy complications

**FIGURE 3:**
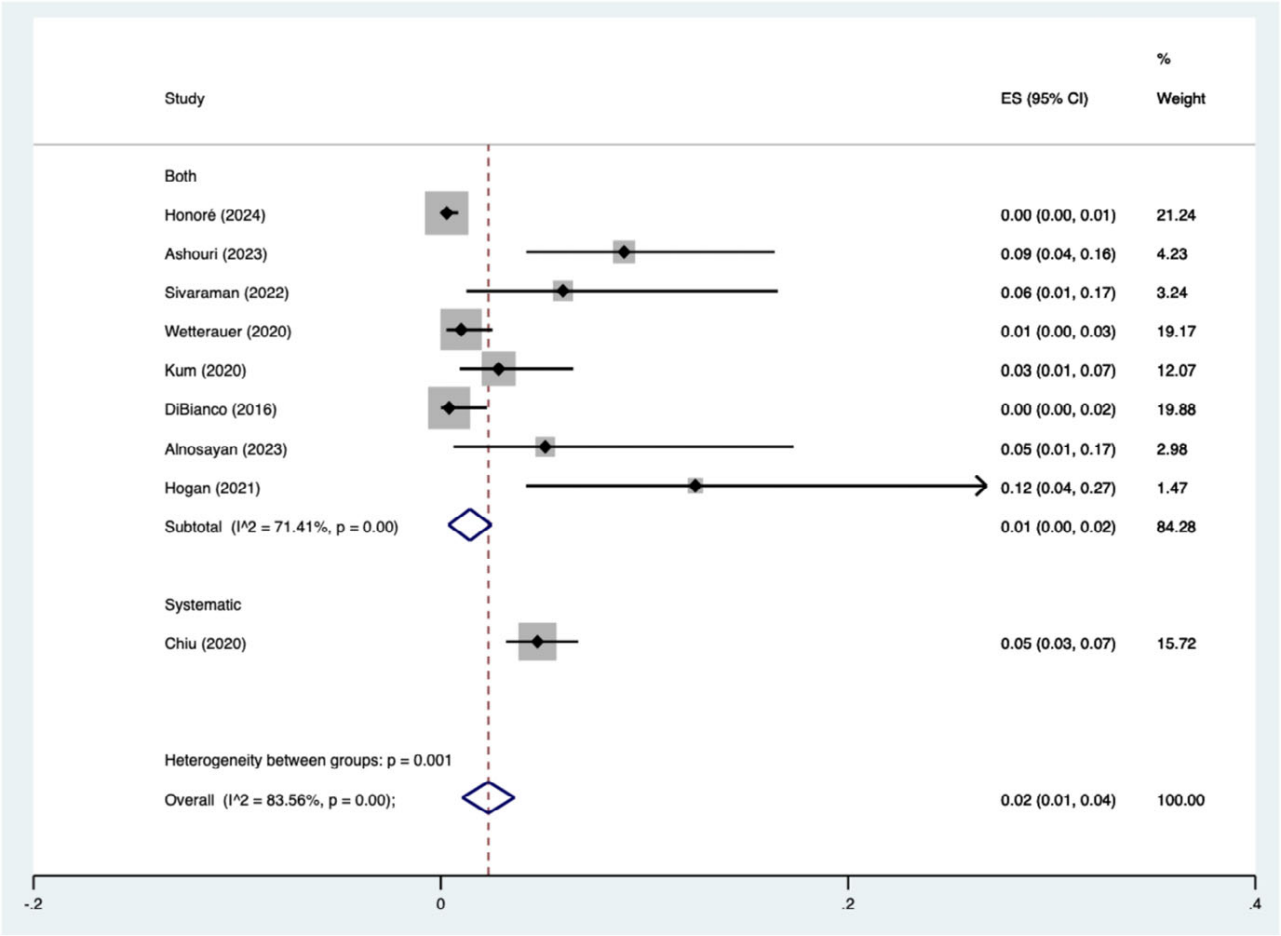
Clavien–Dindo 1 and 2 pooled rates of complications post biopsy.

Complications after prostate biopsies were reported by nine studies with a combined 2688 patients. There were 61 patients (2.3%) who had CD 1–2 complications and three (0.1%) patients who had CD 3–5 complications. All CD 3–5 complications were reported by Ashouri et al.^23^ Our meta‐analysis regarding CD 1 and 2 complications can be observed in Figure 3, showing rates of 2% (95% CI 1–4%). *I* ^2^ was 83.6% and *p* < 0.05.

### 3.6 Secondary outcomes

#### 3.6.1 Pain scores

Pain scores were recorded by nine studies with a combined 1570 patients. Three studies used the numerial rating score (NRS) pain scoring tool. Two reported mean scores of 3 and 2, while one reported a median score of 2. Five used the verbal rating scale (VAS) tool with three reporting median scores of 1, 2 and 2 and two reporting mean scores of 2.1 and 1.8. One used the VAS/100 scoring tool and had a median score of 28. Postbiopsy complications and pain scores are outlined in Table 4. Pain scores were reported during the biopsy procedure and these scores are reported at time of biopsy sampling.

**TABLE 4:** Post biopsy pain and complications.

Author	Clavien–Dindo 1–2	Clavien–Dindo 3–5	Pain scoring tool	Pain score ± SD
Honoré 2024	3 (0.3%)	0	‐	‐
Ashouri 2023	9 (9.0%)	3 (3.0%)	NRS	Median 2 (IQR 0–5)
Silva 2023	‐	‐	NRS	Mean 3 ± 3
Sivaraman 2022	3 (6.0%)	0	VAS	Median 1 (IQR 0–6)
Chiu 2020	29 (4.7%)	0	VAS	Mean 2.1 ± 1.8
Wetterauer 2020	4 (1.0%)	0	VAS	Median 2 (range 0–8)
Kum 2020	5 (2.8%)	0	VAS/100	Median 28 (12.75–52)
Thurtle 2018	‐	‐	VAS	Mean 1.8 ± 1.2
DiBianco 2016	1 (0.4%)	0	‐	‐
Ngu 2023	‐	‐	VAS	Median 2 (IQR 1–3)
Alnosayan 2023	2 (5.1%)	0	‐	‐
Hogan 2021	5 (12.5%)	0	NRS	Mean 2 ± 4

### 3.7 Risk of bias assessment

A risk of bias assessment was performed using a modified Newcastle–Ottawa scale bias tool. Three studies received a score of 7,^2^, ^22^, ^26^ and four studies received a score of 6^4^, ^5^, ^24^, ^29^. Four studies received a score of 5,^23^, ^25^, ^27^, ^30^ and one study received a score of 4.^28^ The full risk of bias assessments is outlined in Supporting Information S1.

## 4 DISCUSSION

This systematic review and meta‐analysis reports the use of TPPB under local anaesthesia with or without sedation using a cognitive fusion or systematic approach. We report rates of 52% (95% CI 44%–60%) for studies that employed both a mix of systematic and targeted biopsies, 26% (95% CI 23%–30%) when systematic biopsies alone are taken and 48% (95% CI 37%–59%) when all data is included. This shows that our data is in line with that of the PREVENT trial which employed a targeted and systematic approach.^8^ Unfortunately, this trial was excluded as some patients may have had their biopsy with the aid of MRI fusion software and this review is specifically examining cognitive fusion; however, it serves as a good reference standard.^35^

From our results, there is no evidence of a statistically significant difference in CSPC in the local anaesthetic TRUS and TPPB cohorts in the literature. In addition, these pooled results indicate that performing this diagnostic procedure under local anaesthetic as opposed to general, does not compromise on CSPC detection rates if we compare our rates versus those when general anaesthesia is used.^2^ We did not, however, directly statistically analyse TPPB under local and general anaesthetic but rather pool the rates under local anaesthesia and compare this to the available literature.

As mentioned, this systematic review and meta‐analysis examined only cognitive fusion or systematic biopsies. This was chosen as the targeting method of choice due to its accessibly over the MRI/US fusion software packages, which many units across the Western world may not have access to at this time.

Additionally, a comprehensive review has suggested that although MRI/US fusion software may have its cancer detection benefits, when combined with systematic biopsy the results between cognitive fusion and software targeted are similar. The cost effectiveness between the two methods was also examined with further study recommended due to the limitations of the study.^36^

One of the main concerns with undertaking this procedure under local anaesthetic is that of pain.^8^ However, as we can observe in Table 4, all mean or median pain scores on the VAS or NRS score are below 3. This can be classified as mild pain.^37^ These synthesised results serve to further support the current evidence that TPPB under local anaesthesia is a well‐tolerated procedure.^8^, ^38^

The complications of the procedure were reported in line with the CD classification.^39^ We can see the procedure was safe with only three adverse events above a CD 2 reported in included studies; the authors did not extrapolate the specifics of each.^23^ It should be noted that all the greater than CD 2 events were reported within the one study. Additionally, the highest rates of CD 1–2 complications were reported by Hogan et al. They had one (2.5%) patient suffer from acute urinary retention requiring a urinary catheter. They also found that recovery time in minutes was 51 for template biopsy under general aesthetic compared with only 18 min for freehand TPPB.^2^ Overall, we can see our pooled rates of CD 1–2 complications is 2% (95% CI 1–4%), which is similar but slightly higher than that found in the PREVENT trial (0.3%).^8^

We postulate that this may be due to refinements in technique within the trial; however, evidence suggests no increased risk of complications in relation to a learning curve.^40^

One should also consider the environmental impact of TPPB under local anaesthesia as the negative effects of gaseous anaesthetic are well documented.^41^ In addition to the clinical and environmental benefits, it has been suggested in the literature that TPPB under local anaesthesia and using US/MRI fusion is more cost effective and safer than the general anaesthetic alternative.^42^

The limitations of this systematic review and meta‐analysis include the retrospective nature of many included studies and the inherent limitations of the studies themselves. The topic of prostate biopsy is a rapidly advancing area, and the current treatment paradigm is constantly evolving with new evidence emerging regularly. Additionally, the true nature of the effect of targeted biopsy is not captured in its entirety as many publications had targeted and nontargeted patient cohorts within the same sample. Robotic‐assisted prostate biopsy has been undertaken under local anaesthetic, and future study should incorporate a meta‐analysis between freehand, MR fusion and robotic biopsy methods in terms of CSPC rates and complications under local anaesthesia.^43^ Additionally, the role of PSMA‐PET in targeting lesions in biopsy naïve high‐risk patients should be further examined.^44^ The role of perilesional targeted biopsy alone may also be examined in further studies.

In conclusion, TPPB under local anaesthesia is a well‐tolerated, clinical procedure with comparable detection rates to other methods and saves the morbidity and logistical issues associated with general anaesthesia.

## AUTHOR CONTRIBUTIONS

BMC: Writing the manuscript, screening, statistical analysis, editing the manuscript; GC: Writing the manuscript, screening, editing the manuscript; JB: Writing the manuscript, data collection; WQ: Writing the manuscript, data collection; AD: Writing the manuscript, data collection; EC: Writing the manuscript, data collection; HT: Editing the manuscript, data collection; RMC: Editing the manuscript, data collection; WS: Supervision, editing the manuscript; LY: Supervision, editing the manuscript; CC: Supervision, editing the manuscript; JK: Supervision, editing the manuscript; PD: Supervision, editing the manuscript.

## CONFLICT OF INTEREST STATEMENT

The authors have no conflict of interest to declare.

## Supporting information


**Data S1.** Supporting Information.
