# Supplementary material for: Transperineal prostate biopsy with freehand technique under local anaesthetic: A systematic review and meta‐analysis
Source: BJUI Compass. 2025 Apr 8;6(4):e70016. doi: 10.1002/bco2.70016 (PMC11977404; doi:10.1002/bco2.70016)
Supplement: Supplementary file 1 — Data S1. Supporting Information. [file BCO2-6-e70016-s001.docx]

| Author | **Selection** | | | | **Comparability** | **Outcome** | | | **Quality** |
| --- | --- | --- | --- | --- | --- | --- | --- | --- | --- |
|  | **Representativeness of the exposed cohort** | **Sample size (<25 = no star)** | **Non-respondents** | **Ascertainment of the exposure** | **The subjects in different outcome groups are comparable** | **Assessment of outcome** | **Statistical test** | **Period (<4weeks)** |  |
| Honore | x | x | - | x | x | x | x | x | 7 |
| Ashouri | x | x | - | x | x | - | - | x | 5 |
| Silva | x | x | - | x | x | x | - | x | 6 |
| Sivaraman | x | x | - | x | x | - | - | x | 5 |
| Chiu | x | x | - | x | x | x | x | x | 7 |
| Wetterauer | x | x | - | X | x | - | - | x | 5 |
| Kum | x | x | - | - | x | - | - | x | 4 |
| Thurtle | x | x | X | X | x | X | - | - | 6 |
| DiBianco | x | x | - | x | x | x | - | x | 6 |
| Ngu | x | x | - | x | x | - | X | X | 6 |
| Alnosayan | x | x | - | - | x | - | X | X | 5 |
| Hogan | x | x | x | x | x | - | X | x | 7 |
